# Closing the gap between implementation science and policy in Nigeria: lessons from the Nigeria implementation science alliance using a nominal group technique

**DOI:** 10.3389/frhs.2025.1629317

**Published:** 2025-11-11

**Authors:** Tonia C. Onyeka, Babayemi Olakunde, Otoyo Toyo, Ijeoma U. Itanyi, Andy Eyo, Dina Patel, John Olawepo, Patrick Dakum, Prosper Okonkwo, Michael Obiefune, John Oko, Bolanle Oyeledun, Ayodotun Olutola, Bola Gobir, Oniyire Adetiloye, Nguavese Torbunde, Muyi Aina, Sidney Sampson, Hamisu Salihu, Joseph Olisa, Vidya Vedham, Mark Parascandola, Patti Gravitt, Gregory A. Aarons, Echezona E. Ezeanolue

**Affiliations:** 1 IVAN Research Institute, Enugu, Nigeria; 2 Department of Anaesthesia/Pain & Palliative Care Unit, College of Medicine, University of Nigeria, Enugu, Nigeria; 3 Department of Population and Community Health, College of Public Health, University of North Texas Health Science Center, Fort Worth, TX, United States; 4 Excellence Community Education Welfare Scheme, Abuja, Nigeria; 5 Department of Community Medicine, University of Nigeria, Enugu, Nigeria; 6 Dalla Lana School of Public Health, University of Toronto, Toronto, ON, Canada; 7 HealthySunrise Foundation, Nevada, NV, United States; 8 Bouvé College of Health Sciences, Northeastern University, Boston, MA, United States; 9 Institute of Human Virology, Abuja, Nigeria; 10 APIN Public Health Initiatives, Abuja, Nigeria; 11 Catholic Caritas Foundation of Nigeria, Abuja, Nigeria; 12 Centre for Integrated Health Programs, Abuja, Nigeria; 13 Center for Clinical Care and Clinical Research, Abuja, Nigeria; 14 Georgetown Global Health Nigeria, Abuja, Nigeria; 15 Jhpiegpo, Abuja, Nigeria; 16 Center for Disease Control, Abuja, Nigeria; 17 Solina, Abuja, Nigeria; 18 Sydani Group, Abuja, Nigeria; 19 Kano Independent Research Centre Trust, Kano, Nigeria; 20 Direct Consulting and Logistics, Abuja, Nigeria; 21 Center for Global Health, National Cancer Institute, Maryland, MD, United States; 22 Department of Psychiatry, University of California, San Diego, CA, United States; 23 Department of Pediatrics, College of Medicine, University of Nigeria, Ituku-Ozalla, Enugu, Nigeria

**Keywords:** implementation science, knowledge translation, policymaker engagement, nominal group technique, collaboration framework, capacity building

## Abstract

**Introduction:**

Knowledge translation in healthcare has been of keen interest to researchers, practitioners, policymakers and administrators as it seeks to confront complex health issues within communities by closing the gap between knowledge generation through research and knowledge application. A paucity of information exists regarding nature of the relationship between Nigerian implementation science researchers and policymakers in the sphere of knowledge translation. This study aimed to identify and discuss barriers to successful engagement between implementation researchers and policymakers as well as to identify strategies for successful engagement between both parties in Nigeria.

**Methods:**

A modified Nominal Group Technique was conducted with 259 diverse health research stakeholders attending the 7th Nigeria Implementation Science Alliance conference in Abuja, Nigeria, to identify barriers to knowledge translation in Nigerian healthcare settings.

**Results:**

Lack of interest in non-aligned priorities of implementation researchers and policymakers, knowledge and capacity gap in stakeholder engagement, and non-existence of engagement framework were ranked as the top three barriers. Developing and sustaining an effective engagement framework, aligning researcher-policymaker interests through collaborative research projects, and joint capacity-building were ranked the topmost facilitators of researcher-policymaker engagement.

**Conclusion:**

This study highlights key barriers to research-to-policy engagement in Nigeria, namely the need for structured engagement frameworks, alignment of priorities, and targeted capacity development, and proposes actionable strategies to address them. Sustainable impact will depend on dedicated financing, governance reforms, and institutional changes, supported by long-term partnerships and robust evaluation systems to advance knowledge translation and improve health outcomes.

## Introduction

In the complex landscape of contemporary policymaking, the imperative to bridge the gap between research and practice has never been more pronounced. As people and communities increasingly face complex healthcare issues such as poor healthcare accessibility, disparities in healthcare outcomes, chronic disease management and the burden of non-communicable illnesses, there has been a remarkable increase in demand for well-supported policies and programs rooted in empirical evidence. Implementation science (IS), a growing field at the intersection of research and practice, has emerged as a vital conduit for translating research findings into actionable policies and interventions (1, 2). Defined as “*the study of methods to promote the integration of research findings and evidence into health care policy and practice to achieve their potential public health impact*” (3), IS provides tools for closing the evidence-to-policy gap. Effective knowledge translation hinges on the premise that research should be relevant, accessible, and influential in shaping policies and practices. Policymakers and practitioners require timely access to evidence to inform their decisions, while researchers yearn for their findings to have a real-world impact (4). Actualizing these potentials require effective engagement, especially between policymakers and implementation science researchers (5), and the ability to navigate the intricate dynamics of their diverse settings (6). Successful knowledge translation is therefore not straightforward; it requires deliberate and systematic efforts to engage relevant stakeholders so that research findings resonate with their needs, values, and constraints (6).

The health policy landscape in Nigeria is unstable due to a mix of political, institutional, and social factors, which often complicate the implementation of policies (7). For instance, while the 2014 National Health Act was a significant reform aimed at improving the uptake of public healthcare services, limited funding commitment for universal health coverage has inadvertently reinforced private sector dominance (8). Other major initiatives such as the National Health Act and the Basic Health Care Provision Fund have also experienced chaotic implementation, including fragmented authority, frequent political turnover, and unreliable financial flows (7, 9).

When researchers and policymakers jointly design, conduct, and apply research, this promotes ownership and uptake, an approach critical for closing the research-implementation gap and making findings more relevant to policy needs (10–12). In Canada and Europe, the widely used integrated knowledge translation approach reduces power imbalances between researchers and policymakers and makes both parties equal partners throughout the research process, from question development to dissemination (13, 14). This study looks at the nature of engagement between Nigerian IS researchers and policymakers in the broader context of knowledge translation. Specifically, it identifies barriers and proposes strategies to promote effective collaboration between these two groups of stakeholders. While previous studies have demonstrated the value of engagement, describing it as reciprocal and relational, involving trust, mutual respect, and open communication (15, 16), there is limited evidence on how this plays out within Nigeria's health policy environment. Engagement brings stakeholders, including research participants, citizens, and policymakers, closer to the research process, ensuring that outcomes are aligned with their needs (17, 18). In the field of IS, such engagement is essential for ensuring the credibility, relevance, and applicability of research findings (18, 19). The NIH-PEPFAR PMTCT Implementation Science Alliance is a good example of how researcher–policymaker partnerships can strengthen communication and enhance community-based interventions (20, 21). Yet, despite such models, collaboration in the Nigerian context remains fragmented and limited (2, 22). While efforts such as co-designed research, knowledge transfer, and evidence-informed decision-making have shown promise (5, 23), the attempts were limited to only a few states and may not reflect the national picture. Also, few studies have critically studied the engagement process from the perspective of implementation researchers themselves, professionals who operate at the intersection of evidence generation and real-world application. Existing research on knowledge translation in the Nigerian context has largely focused on general strategies for getting research into policy and practice, with limited attention to the voices, experiences, needs and insights of IS scientists themselves. This study addresses that gap by drawing attention to their perspectives to offer context-sensitive insights into how meaningful engagement can be promoted. By identifying both systemic and relational challenges, this study aims to place a spotlight on the conditions necessary for successful and sustained policymaker–researcher partnerships in Nigeria. These contributions are particularly timely in light of Nigeria's ongoing health reforms and the increasing emphasis on evidence-informed policy-making, which demand more integrated, cross-sector collaboration.

## Methods

### Study design and population

We conducted a modified version of the Nominal Group Technique (NGT) during the two-day Conference of the Nigeria Implementation Science Alliance (NISA) held from August 17th to 18th, 2023, in Abuja, Nigeria, titled under the theme “Bridging the Implementation Science Gap: Strategies for Effectively Translating Evidence into Practice.” The NGT, a structured, face-to-face group brainstorming method, was selected for its efficacy in collecting and establishing consensus on diverse issues, challenges, and solutions among a heterogenous group of stakeholders (24, 25). Its suitability for time-limited, in-person engagement with diverse groups, and its ability to combine the strengths of both quantitative and qualitative approaches by allowing for collective prioritization through consensus and ranking and the inclusion of rich contexts, made it an ideal choice. This study utilized a modified NGT, incorporating hybrid voting platforms, purposive assignment of participants to tables, virtual documentation of barriers and facilitators using Microsoft Forms (as opposed to traditional paper) and a two-day phased process for consolidation and plenary voting (24, 25). The NGT process is particularly advantageous as it ensures equitable participation, thereby mitigating the dominance of more vocal individuals (17). This advantage was particularly important given the diversity of expertise among implementation science researchers and the need to surface and rank the most context-relevant barriers and strategies. Compared to the Delphi method, which requires multiple rounds of remote input over time, and Focus group discussions (FGDs) and interviews that limit the scope of perspectives, the NGT methodology was deemed to be the most suitable approach as it would result in the elicitation of practical, context-specific solutions from conference attendees, relying on their firsthand experiences, to identify and rate key challenges and actionable strategies in a time-limited group session.

Eligible participants were adult attendees of the 2023 NISA conference who identified as researchers, policymakers, implementing partners, civil society actors, or affiliated stakeholders in implementation science or public policy. Inclusion criteria included self-identification with one of these professional categories during the conference registration process and willingness to participate in the group session. There were no exclusion criteria aside from non-attendance or withdrawal of consent. Participants were purposively allocated to 32 tables using registration data collected prior to the conference. Allocation was stratified by stakeholder category (e.g., policymaker, researcher, implementer) as self-identified at registration, in order to ensure heterogeneity within each table and to maximize cross-sectoral perspectives. The assignment process aimed to ensure a balanced and representative mix of disciplines at each table, reduce the risk of stakeholder dominance and increase diversity of perspectives. Thus, by integrating a wide spectrum of voices (across government, academia, and practice) into each group, we aimed to minimize sample bias thus ensuring a more comprehensive consensus-building.

### Procedure

**Figure 1:**
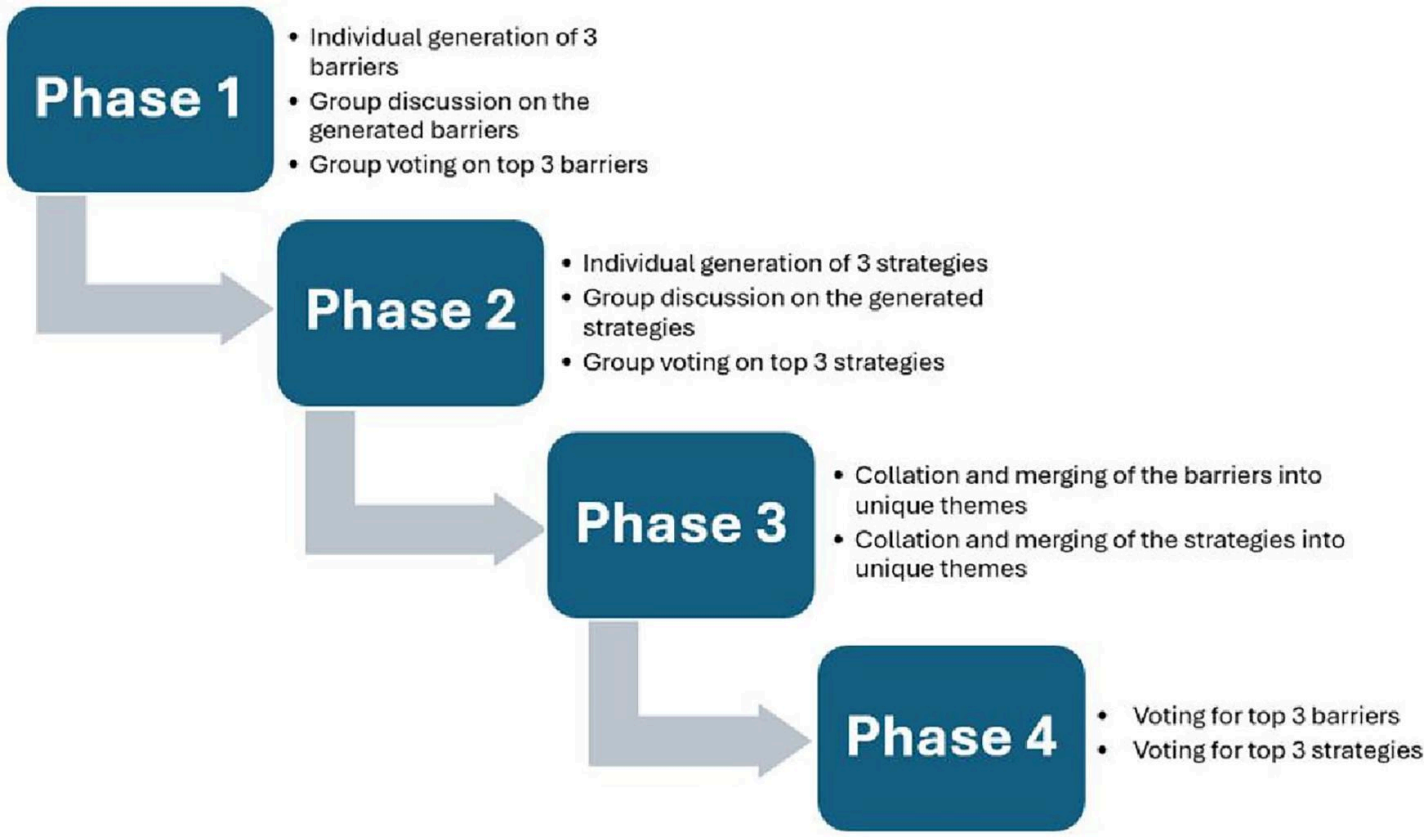
NGT procedure.

The NGT session was led by four trained facilitators and followed a hybrid model, combining in-person and virtual tools. Each group had a designated leader who was provided with a Samsung Galaxy Tablet® (10.1″) and standardized instructions. The session began with a brief presentation on the purpose and structure of the NGT. Participants were at their assigned tables (Table numbers 1–32), which had been pre-arranged to ensure stakeholder diversity as described above. The NGT unfolded in four phases across two days and comprised four distinct phases as illustrated in Figure 1. *Phase 1* was dedicated to identifying barriers that hinder successful engagement between policymakers and implementation researchers in knowledge translation. This phase encompassed three stages. The first stage was idea generation, where individual participants listed their top three barriers to successful engagement between policymakers and implementation researchers for knowledge translation. In the group discussion stage, the barriers generated by individual participants were thoroughly discussed within each group. In the voting stage, group members subsequently identified and voted for the top three barriers that had emerged from their discussions.

Moving on to *Phase 2* of the NGT, participants focused on pinpointing the three most promising strategies to address each of the top three barriers identified within their respective groups. As in Phase 1, this phase followed a structured approach, comprising idea generation, discussion, and voting. In *Phase 3*, the facilitators amalgamated the top three barriers identified by all 32 groups, de-duplicating before consolidating them into cohesive themes. Proposed strategies for each group's top three barriers were also merged into unique strategy themes following de-duplication. All three phases happened on Day 1. *Phase 4* involved a plenary session in which participants first voted for the top three barriers identified from the themes generated in Phase 3. They then voted for the top three strategies to address each of these barriers. Phases 1 and 2 of the NGT together lasted approximately 1 h and 20 min. During Phase 4, voting on barriers lasted about 7 min, while voting on corresponding strategies took about 20 min. Both voting activities were conducted on Day 2 of the conference (see Supplementary Table). Minimal attrition (<3%) occurred during Phase 4 plenary voting, due to a few participants leaving the conference early and a brief period of technical/connectivity issues. Only complete voting records were analysed, consistent with NGT data handling standards, with no partial votes included.

### Data collection

Barriers and strategies were recorded via Microsoft Forms with QR codes for rapid consolidation.. During Phase 1 and 2, the voting procedure took a digital form, utilizing OpinionX© (https://www.opinionx.co) for collating group barriers and strategies while Phase 4 utilized Mentimeter© (https://www.mentimeter.com), an external online platform tailored for virtual interactive polling and ranking, to deliberate on strategies. OpinionX© was incorporated for barrier ranking due to its ability to handle a high number of options, and Mentimeter© for strategy ranking, which allowed for interactive visualisation and quick consensus (both being a deviation from traditional in-room card or flipchart voting). Facilitators generated QR codes linked to the polls that were then projected onto a screen for participants to access. Responses were only unveiled after all participants had completed their voting.

### Analysis

The analysis process adhered to the prescribed methodology for handling NGT data, as outlined in the guidelines proposed by McMillan and colleagues (24). To examine the barriers and potential strategies facilitating productive collaboration between policymakers and implementation researchers in diverse contexts, a thematic analysis approach was applied. This involved the aggregation and categorization of all valid responses, which were then grouped according to common themes. Descriptive statistics, including frequencies and percentages, were employed to provide a concise summary of the voting outcomes.

### Ethical considerations

This study involved human subjects. However, the need for ethical approval for this study was waived by the College of Medicine Research and Ethics Committee (COMREC), University of Nigeria, as the research did not involve interventions or collection of sensitive personal data that required review, according to institutional guidelines. All participants were provided with clear information about the purpose, procedures, and voluntary nature of the study both before and during the NGT session. Informed consent was obtained verbally from all participants, and participation in the NGT activities implied consent to contribute data anonymously to the study. No identifying information was collected during idea generation, discussion, or voting phases. Data were stored in password-protected files and accessible only to the research team to ensure confidentiality and data protection. Given that this activity was conducted during a professional conference, and responses were aggregated at the group level, the risk to participants was minimal. Participants had the right to decline participation at any time during the process without consequence.

## Results

A total of 259 individuals (Males: 177; Females: 82) participated in the NGT session, comprising 189 implementation practitioners, 53 researchers, 11 policymakers and 6 healthcare providers. The average group size was 8 persons. Table 1 presents the demographic distribution of the participants.

**Table 1:** Demographic distribution of respondents.

Participant type	Number of participants	Percentage (%)
Gender		
Male	177	68.33
Female	82	31.66
Role type		
Implementation practitioners	189	72.97
Researchers	53	20.46
Healthcare workers	6	2.32
Policy makers	11	4.35

### Identified challenges/barriers

**Figure 2:**
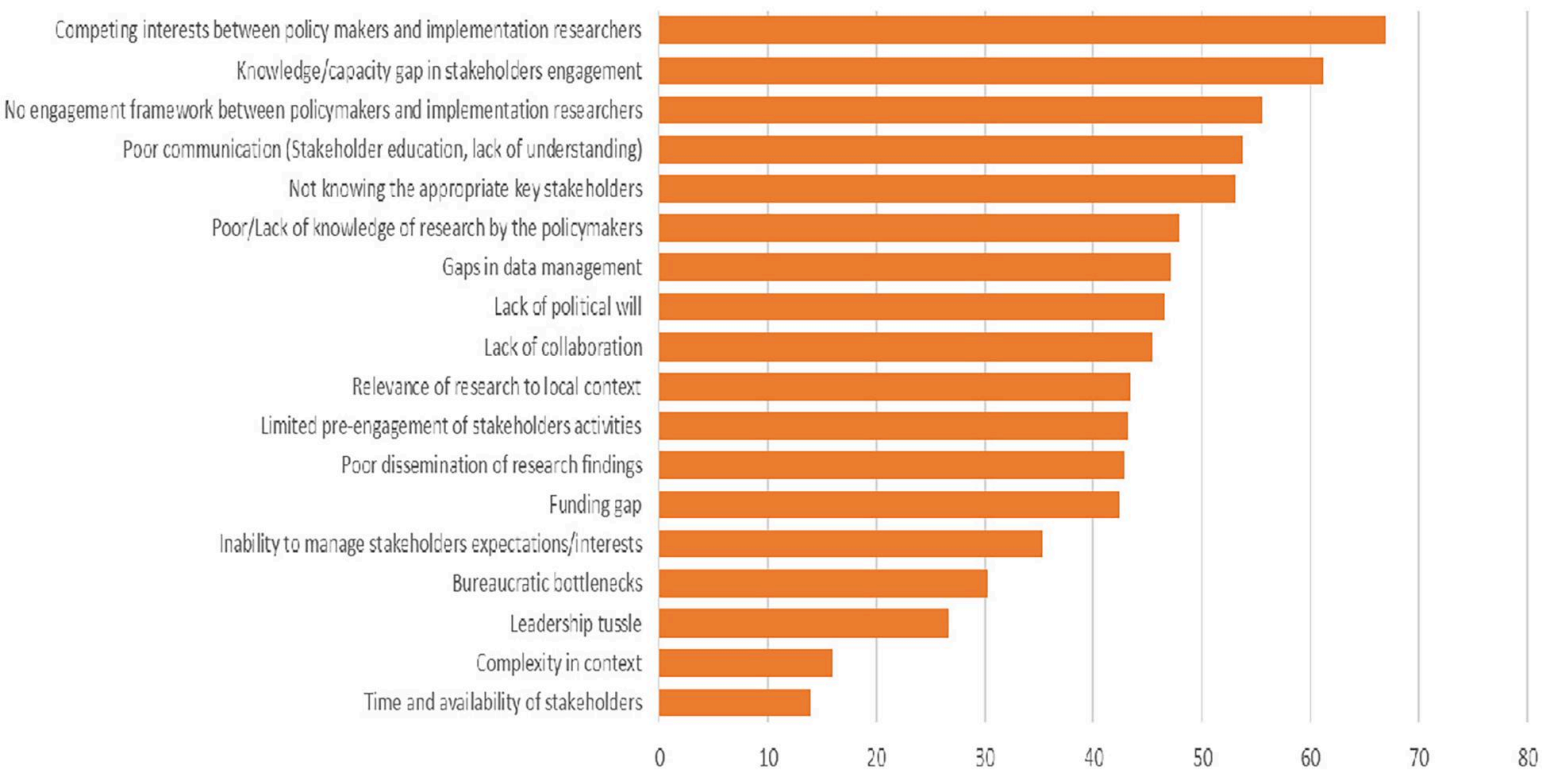
Ranking of unique barriers using OpinionX.

Following de-duplication, one hundred and eighty-one (181) unique responses were received from the groups during *Phase 1* of the NGT, with each group contributing a minimum of between three to five barriers. These barriers were categorized under themes during the collation process. The themes identified include “knowledge/capacity gaps in stakeholder engagement”, “lack of an engagement framework between policymakers and implementation researchers”, “effects of the political environment and bureaucracy”, “competing interests”, “lack of resources”, “communication gaps”, “lack of collaboration”, “the lack of relevance of research findings to the local context”, “absence of dissemination opportunities”, etc. Eighteen (18) unique barrier themes were identified and voted during *Phase 4* of the NGT process as seen in Figure 2. “Competing interests between policymakers and implementation researchers” emerged as the highest-ranked barrier, accounting for 28% of collated votes. This was followed by “knowledge/capacity gap in stakeholder engagement”, and “absence of an engagement framework between policymakers and implementation researchers”, in that order. Conversely, “lack of time and availability of stakeholders”, “complexity of the research”, and “leadership tussle in implementation” were ranked as the least relevant barriers.

### Identified strategies for successful engagement

**Figure 3:**
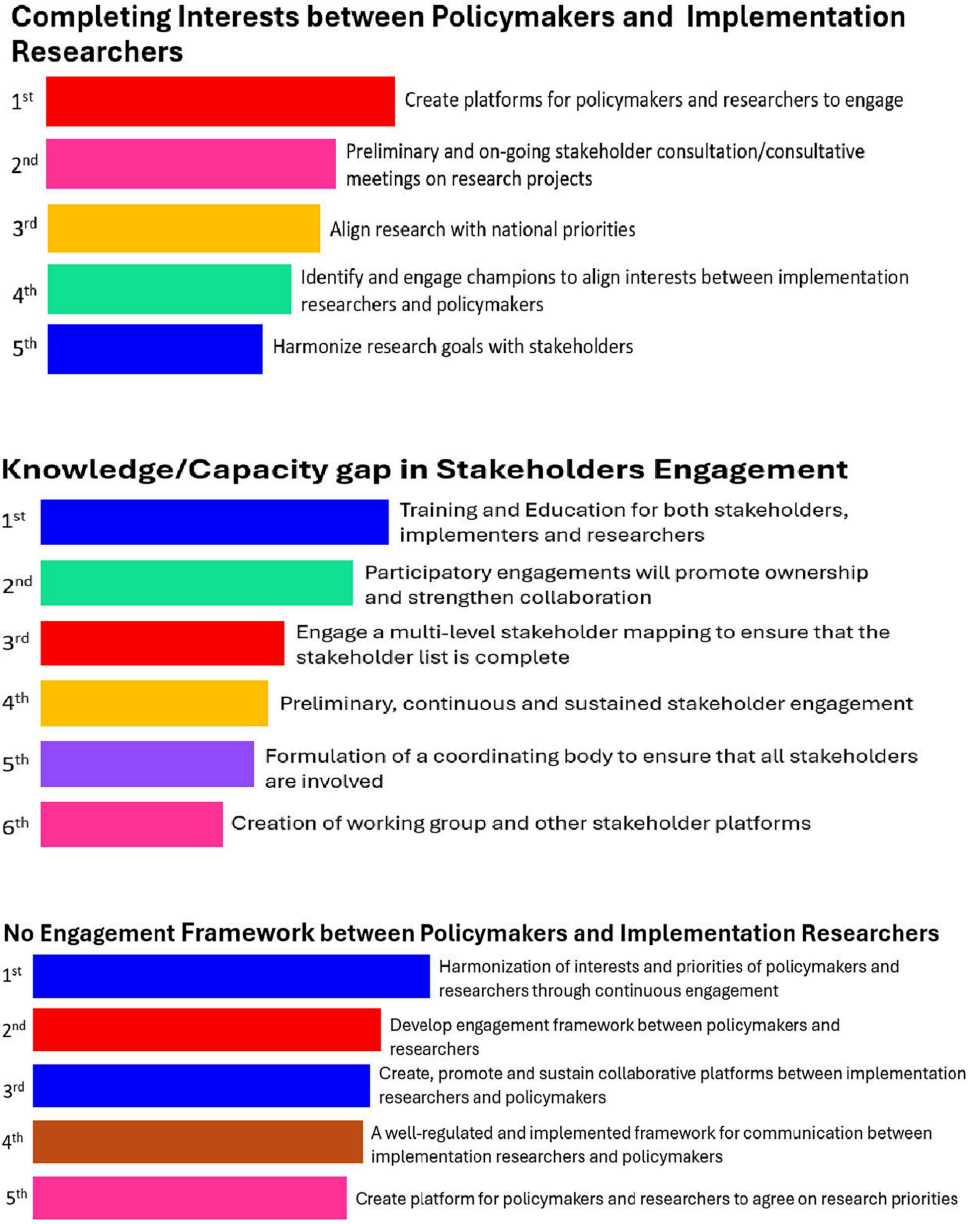
Output from mentimeter on topmost strategies explored to address topmost 3 barriers for successful engagement between policymakers and implementation researchers.

Eighty-one (81) responses were received from the groups during Phase 1 of the NGT, for each of the top three listed barriers. Participants highlighted creating “a platform for researchers and policymakers to engage”, “stakeholder consultation meetings on research projects” and “aligning research with national priorities” as the top three strategies for successful engagement between policymakers and implementation researchers. Strategies to overcome the “knowledge/capacity gap in stakeholder engagement” from the NGT process include “training and education of stakeholders”, “participatory engagements”, and “multi-level and complete stakeholder mapping”. To overcome the “absence of an engagement framework between policymakers and implementation researchers”, participants listed “continuous engagement to harmonize the interests and priorities of researchers and policymakers”, “developing an engagement framework between policymakers and researchers”, and “creating or sustaining collaborative platforms between researchers and policymakers” as the top three most effective strategies (Figure 3). It should be noted that outputs presented in Figures 2, 3 reflect relative ranking rather than scale-based measurements.

## Discussion

In this NGT exercise, participants identified three top themes related to the barriers to successful engagement between implementation researchers and policymakers: (1) the absence of a structured engagement framework, (2) competing interests between the two groups, and (3) knowledge and capacity gaps in stakeholder engagement. These findings resonate with similar challenges reported in the literature and can be meaningfully understood through the lens of implementation and policy translation theories and frameworks.

### Barrier 1: absence of a structured engagement framework

This barrier mentioned by conference participants reflects a fragmented research–policy–practice interface, which, according to the EPIS framework, constitutes a failure of systems-level bridging mechanisms and organizational readiness and sustainment strategies (26). In the Nigerian context, which is similar to many low- and middle-income countries (LMICs), institutional fragility and limited absorptive capacity further complicate the seamless integration of research into policy. This challenge mirrors findings in the literature. For example, a study in the Benin Republic revealed a clear disconnect between research evidence and policy formulation in developing the user fee exemption policy for caesarean sections (CS), the latter being part of some electoral campaign promises made good by a prominent Beninois politician (27). Although public health evidence could have steered the policy toward reducing maternal mortality, its political goal was instead to end the detention of mothers and newborns unable to pay hospital fees by exempting them from a broad package of maternal health services., (the césarienne gratuite or free CS policy) (27). In the end, the policy was unable to achieve free CS for all and, unfortunately, made little or no impact in the reduction of maternal and neonatal morbidity and mortality.

This narrative above illustrates what the Knowledge-to-Action (KTA) framework identifies as a breakdown in adapting knowledge to the local context and a failure to assess barriers to its use (28–30). Within this model, stakeholder engagement is a dynamic and iterative process, requiring both capacity and mutual understanding, elements perceived by study participants to be lacking. The absence of such competencies on both sides (research literacy among policymakers and policy literacy among researchers) hampers the KTA cycle, effectively stalling knowledge translation into policy and practice. Bullock et al. (31) theorize that implementation science and policy implementation research have often evolved separately, creating theoretical gaps and limiting integration of policy perspectives in implementation frameworks. In other words, policy is often treated as context or a barrier rather than as an active process involving key actors, leading to insufficient integration of policymakers in implementation efforts. Crable et al. (2) further argue that most dissemination and implementation (D&I) frameworks lack explicit guidance for how and when to engage policymakers. In addition, limited awareness of D&I frameworks among policymakers often compounds the problem, further hindering rigorous evaluation and adaptation of health interventions (32). This gap is particularly problematic in LMICs like Nigeria, where fragile health systems demand not just innovative interventions but also timely and politically feasible implementation strategies. Consequently, scientific advances remain trapped within academic or pilot phases. Without intentional mechanisms to bridge this divide, engagement will remain fragmented and superficial, preventing evidence from influencing meaningful policy action (33).

#### Proposed strategies

Harmonisation of the interests and priorities of policymakers and researchers through continuous engagement, development of an engagement framework between researchers and policymakers, and the creation, promotion, and sustainment of collaborations between the two groups were suggestions raised by participants to eliminate this barrier. One suggested way to address this problem is by rethinking how research questions are defined, using a collaborative, co-creation approach. Sienkiewicz (34) posits that it is more beneficial for researchers and policymakers to co-create the research questions early in the process, and make attempts to translate policy issues into research questions, thus increasing the value of policy-relevant research (35). To enhance the researcher-policymaker collaboration, the Boyer's model of engaged scholarship emphasizes that researchers should collaborate with policymakers and communities in defining research questions (co-creation), integrate academic knowledge with practical and policy contexts, ensure that research findings are translated into accessible, policy-relevant outputs and work in an ongoing, two-way relationship where both scholars and policymakers shape and benefit from the process (36). Another way to ensure harmonisation of interests and priorities between researchers and policymakers is through exchange programs. The European Commission's Joint Research Centre (JRC) has a short-exchange program that encourages researchers to have short stints of 2–4 weeks in policy departments relevant to their research interests (34). The exchange aims to enhance scientists' understanding of policy processes, build strategic collaborations, provide input on key policy issues, and offer targeted support on scientific matters in policy debates.

### Barrier 2: competing interests between researchers and policymakers

The second major barrier, which was mentioned by conference participants to be competing interests, can arise because policymakers often prioritize political feasibility, resource constraints, and rapid decision-making, whereas researchers emphasize methodological rigor, evidence generation, and long-term outcomes, leading to potential misalignment in goals and timelines (2, 31). This divergence can be explained using the Stakeholder theory, which posits that different actors within a system hold distinct, and sometimes conflicting, stakes in decision-making processes (37, 38). Without deliberate mechanisms to reconcile these differences, collaboration becomes superficial or counterproductive. This barrier is also known to be exacerbated by asymmetry in capacity-building opportunities. For instance, a mapping study found most training and skills initiatives were skewed towards researchers (39). This challenge mirrors challenges of divergent interests and timelines previously identified in a study by Erismann et al. (40). Similarly, poor collaboration between researchers and practitioners has been reported at a similar gathering as a barrier to evidence-based policymaking in Nigeria (41).

#### Proposed strategies

Participants recommended creating platforms for engagement, holding stakeholder consultation meetings, and aligning research with national priorities. Participatory action research methods have been proposed as a strategy to create structured platforms for collaboration (41). For instance, hybrid effectiveness–implementation research designs which simultaneously evaluate intervention impact and implementation processes, offer dual benefits by linking evidence generation with real-world execution (42, 43). This creates a methodological “meeting point” where researchers' evidence priorities and policymakers' implementation needs converge, enabling both groups to act on results more quickly and effectively. Such approaches have been successfully applied in Nigeria in domains like mental health and hypertension interventions (44, 45). Jessani et al. (46) further identify key facilitators that strengthen collaboration between researchers and policymakers. These fall into two broad categories: individual-level facilitators that include acquiring knowledge of the policy-making process, understanding stakeholder networks, gaining access to decision-makers, and receiving communication skills training, as well as institutional-level facilitators which include academic encouragement for policy engagement, research-funding guidance to align inquiry with policy needs, and synchronization between academic research timelines and legislative cycles (46). Ensuring that research is aligned with national priorities is important as it will help address the decades-long problem of neglect of research evidence by policymakers and decision-makers. To achieve this, the Nigeria Implementation Science Alliance (NISA) provides a key dissemination and engagement platform that brings together policymakers, implementation practitioners, and researchers to share lessons from public health programs in Nigeria (26). Through its annual conference, NISA fosters collaboration, information exchange, and problem-solving, while supporting the development of culturally-appropriate engagement frameworks. Its diverse stakeholder base enables networking, partnerships, and mutual feedback, ensuring research remains relevant and applicable to policy needs. Sustained by local funding since 2015, NISA's platform has strengthened health research capacity and promoted lasting researcher–policymaker engagement.

The Research-to-Policy Collaboration (RPC) model (22) offers another example. The process for this collaboration framework model begins with researchers identifying policy-relevant areas of their work that address pressing societal issues. Next, the coordinator guides them through a six-week Training and Coaching program of instructional webinars and live practice sessions to build engagement skills. This is followed by a Legislative Needs Assessment with policymakers. During the Rapid Response Event, researchers connect with policymakers, agencies, and stakeholders to understand priorities, create action plans, and develop deliverables such as policy briefs. The final step, Ongoing Collaboration, enables researchers to work more independently with policymakers while continuing to refine their policy competencies under the coordinator's guidance.

Two notable research initiatives in Africa have utilized the Evidence-Informed Decision-Making (EIDM) framework to engage both policymakers and researchers. The Thanzi Programme has cultivated research-to-policy partnerships to support evidence-informed health resource allocation decisions in Malawi, Uganda, and Zambia (47). This has been achieved by the institutionalization of Health Economics and Policy Units, that serve as bridges between ministries of health and universities in those countries. The Health Policy Research Group (HPRG) at the University of Nigeria implements a model that facilitates direct engagement between researchers and policymakers, emphasizing the collaborative processes and addressing challenges like communication gaps and limited policymaker capacity for research uptake (5). The Implementation-STakeholder Engagement Model (I-STEM) (48), has been suggested. It encourages involvement of all stakeholders throughout the implementation process (barrier identification and prioritization, implementation strategy selection and planning, delivery, evaluation, and reporting of stakeholder engagement activities), can be used alongside other theories and frameworks, but will require validation in many contexts. Other methodologies, such as the Systems Analysis and Improvement Approach (SAIA) (49) and the Integrative Systems Praxis for Implementation Research (INSPIRE) (50) also offer structured mechanisms for integrating policy engagement into research practice.

### Barrier 3: knowledge and capacity gaps in stakeholder engagement

Persistent gaps in policy literacy among researchers and research literacy among policymakers emerged as a major barrier mentioned by conference participants. The SPIRIT Action Framework (Supporting Policy In health with Research: an Intervention Trial) (51) is a conceptual model, first introduced in 2015, that helps guide and test strategies to increase the use of research in policymaking. At its core, the framework outlines a logical sequence of the following: a Catalyst which is a factor that triggers the demand or need for policy research (e.g., a pressing policy question or emerging public health issue); Capacity, which is the ability of an organization or individual to engage with research; Actions: which are the research-related behaviours that follow (e.g., accessing, appraising, generating, or interacting around research) and Outcomes which refers to the forms of research use in policymaking (e.g., agenda-setting) and longer term impacts. Within the SPIRIT Action Framework, this barrier is identified fall under the “capacity” domain, which are skills, knowledge, systems, and relationships required for effective research–policy engagement. Such capacity gaps limit progression from catalyst to action, stalling translation of evidence into policy. Similar emphasis on capacity appears in other Knowledge Translation frameworks, such as the Knowledge-to-Action (KTA) cycle (52) and the Capability, Opportunity and Motivation- Behaviour (COM-B) model (53), which frame capability as a prerequisite for behaviour change in evidence use. Without targeted strategies to strengthen mutual understanding and stakeholder engagement skills, interactions can become inefficient and burdensome, with researchers spending disproportionate time lobbying policymakers rather than co-producing policy-relevant evidence.

#### Proposed strategies

Participants raised the top 3 strategies for this barrier to be training and education for both researchers and policymakers, participatory engagements to promote ownership and strengthen collaboration, and use of a multi-level stakeholder mapping to ensure that the stakeholder list includes all relevant parties. Enhancing the linkages between researchers and policymakers can be achieved by having researchers cultivate both technical skills, such as a grasp of the policy-making process, and interpersonal abilities, such as building rapport, both of which can be honed through training opportunities that offer a blend of direct instruction and hands-on experiential learning (22). This training should also include training on how policymakers utilize research as well as their decision-making realities (22), leading to the development of policy competencies. Apprenticeship opportunities in policymaking for researchers who get paired with policy mentors, enrolment in policy programs at university-based policy centers, inclusion of policy coursework and practica within relevant postgraduate curriculum, funding of policy-related research work, and collaboration with policy advocacy organizations are some of the ways to ensure researchers are immersed in policy training opportunities (22, 54). Also, formal training on participatory research and stakeholder engagement should be incorporated into public health training curricula. Community-based participatory research (CBPR) has been developed as a collaborative approach for research that provides local capacity building (55, 56). CBPR contributes to informed decision-making by generating data and insights directly from the community, thus providing policymakers with a more nuanced understanding of local issues and potential solutions. CBPR empowers communities to actively participate in the research process, fostering a sense of ownership. This engagement can lead to more effective policies that align with the community's priorities. Also, policymakers may find CBPR results more relevant and applicable to the specific context of the community, increasing the likelihood of successful policy implementation. The collaborative nature of CBPR encourages joint problem-solving, encouraging policymakers involved in the process to work more closely with communities to develop policies that address identified needs. CBPR builds trust between researchers, community members, and policymakers. When policymakers are involved in the research process, it can enhance their credibility and trustworthiness in the eyes of the community. Successful CBPR implementation, exemplified in Nigeria (57, 58) and similar settings in sub-Saharan Africa (59, 60), is widespread. Other stakeholder engagement methods, such as participatory impact pathways analysis, have been shown to improve collaboration between stakeholders in Uganda (61).

While our NGT recommendations (a tailored engagement framework, harmonization of researcher–policymaker priorities, and targeted capacity strengthening) are evidence-informed and grounded in stakeholder input, their adoption in Nigeria must be judged against entrenched political and governance realities identified in the literature and echoed in our findings. The NGT exercise produced these three top themes and proposed strategies, but Nigeria's policy environment is characterized by strong actor interests, patronage dynamics, frequent leadership turnover, and multi-level fragmentation between federal, state and local actors, conditions that shape which policies are adopted and sustained. Empirical analyses of the Nigerian policy arena show that ideologies, vested interests and institutional incentives often supersede technical evidence in policy choices, and that evidence–policy conflict is a recurrent theme across African health systems (62). In addition, financing and governance constraints [notably the mixed experience with the Basic Health Care Provision Fund (BHCPF) and other pooled financing mechanisms] limit the government's absorptive capacity for new institutional arrangements and for sustainably financing KT (knowledge-to-policy) functions (63). Systematic reviews of scale-up further show that human resources, financing, supply chains and changes in the policy environment are frequent, practical barriers to taking pilot interventions to national scale (64). Taken together, these realities imply our strategies are conditionally feasible. In other word, they can be realistic and scalable only if packaged as politically-attuned, incremental steps (for example, anchoring pilots within existing financing windows such as BHCPF or donor co-financing, identifying and resourcing policy champions, aligning activity timelines to legislative and budget cycles, and building modest, measurable KT outputs into routine ministry reporting). Guidance on applied political analysis and staged scale-up supports these pragmatic approaches (65).

A strength of this study is the direct involvement of key stakeholders, implementation science researchers, policymakers, and practitioners, in collaboratively identifying barriers and potential strategies. Also, integrating an online modification of the Nominal Group Technique (NGT) using platforms such as Mentimeter© and OpinionX© enabled prompt consensus-building and real-time data manipulation. Prior research robustly supports the feasibility of fully online or virtual NGT (vNGT) processes. For example, a multi-country palliative care study found online NGT using Zoom and Mentimeter to be feasible and potentially advantageous compared to in-person sessions (66). A recent scoping review of vNGT in healthcare research similarly identified benefits such as inclusion of geographically dispersed participants, scheduling flexibility, and cost savings (67). These findings reinforce the practicality and strategic value of conducting NGT virtually, particularly when participant numbers are large (e.g., *N* = 259), making timely in-person consensus unlikely within tight conference constraints. While the specific use of OpinionX© in NGT ranking may be novel, given limited published reports, this possibility should be framed cautiously as potentially one of the first documented usages rather than as a confirmed first-ever instance. Limitations of the study include its focus solely on NISA conference attendees from 2023, which may limit broader generalizability across all Nigerian implementation researchers, policymakers, and practitioners. Additionally, as with any NGT, the process may not capture the full depth of individual perspectives. To mitigate this, small breakout groups and personalized virtual forms were used to gather more nuanced views. Future studies could incorporate focus group discussions to promote deeper reflective engagement. Despite these limitations, the findings yield valuable insights to inform strategies for narrowing the implementation research–practice gap.

## Conclusion

Using a national Nominal Group Technique with implementation researchers, policymakers and practitioners, participants identified three primary barriers to effective research-to-policy engagement in Nigeria: (1) the absence of a structured engagement framework; (2) competing interests and misaligned timelines between researchers and policymakers; and (3) persistent knowledge and capacity gaps for stakeholder engagement. The highest-ranked strategies were co-creation of policy-relevant research (early researcher-policymaker question co-definition), creation and institutionalization of a structured engagement framework/platform (to harmonize priorities), and targeted capacity development (training, exchanges and embedded mentorship). These findings emphasize the need for a tailored engagement framework, alignment of interests through collaborative projects, and stakeholder capacity development. Collaboration is crucial, especially as funders now expect research proposals to include strategies to influence policymakers. Moreso, the COVID 19 pandemic has made policymakers more ready than ever to engage with researchers (68). However, researchers must remain vigilant against risks such as loss of objectivity and undue policymaker influence (69).

The study's findings suggest that institutionalizing research-to-policy linkages in Nigeria requires sustainable financing for KT functions such as policy engagement, rapid evidence synthesis, and embedded fellowships, included in national and subnational budgets or tied to pooled funds like the BHCPF, with predictable domestic financing and stronger governance as emphasized by the Lancet Nigeria Commission and PHC financing evaluations (70, 71). Governance reforms that enhance transparency, reduce political interference, and stabilize managerial stewardship are essential to ensure engagement frameworks survive beyond short political cycles, with the BHCPF governance literature providing a cautionary example (63). In addition, institutional reforms in ministries and academia such as establishing evidence units, mandating KT plans in publicly-funded research, and valuing translational outputs, combined with lessons from African partnership models like Thanzi, should be embedded within monitoring and evaluation systems to adapt engagement platforms to political and fiscal realities (47, 72). Future efforts should focus on long-term partnerships, capacity-building, and contextual evaluation mechanisms to ensure sustained, impactful engagement and improve health outcomes through knowledge translation (39). Addressing barriers mentioned and implementing the highlighted strategies holds immense promise in fostering a more cohesive and impactful relationship between policymakers and researchers, ultimately advancing the crucial goal of knowledge translation within Nigerian healthcare.

## Data Availability

The raw data supporting the conclusions of this article will be made available by the authors, without undue reservation.
