# Supplementary material for: Closing the gap between implementation science and policy in Nigeria: lessons from the Nigeria implementation science alliance using a nominal group technique
Source: Front Health Serv. 2025 Nov 11;5:1629317. doi: 10.3389/frhs.2025.1629317 (PMC12644075; doi:10.3389/frhs.2025.1629317)
Supplement: Supplementary file 1 [file Table1.docx]

**Phases of the Nominal Group Technique (NGT) with Time frames and Dates**

| **PHASE** | **ACTIVITY** | **DAY/DATE** | **START TIME** | **END TIME** | **DURATION** |
| --- | --- | --- | --- | --- | --- |
| Phase 1 | Identification of barriers (idea generation, group discussion, voting) | Day 1 – November 17, 2023 | 2.45pm | 3.35pm | 40 minutes |
| Phase 2 | Identification of strategies (idea generation, group discussion, voting) | Day 1 – November 17, 2023 | 3.35pm | 4.15pm | 40 minutes |
| Phase 3 | De-duplication of ideas and consolidation of themes (facilitator team) | Day 1 – November 17, 2023 | 4.15pm | 4.45pm | 30 minutes |
| Phase 4a | Plenary voting by Mentimeter^©^ on top three barriers | Day – November 18, 2023 | 10am | 10.07am | 7 minutes |
| Phase 4b | Plenary voting Mentimeter^©^ on top three strategies per identified barrier | Day 1 – November 18, 2023 | 10.07am | 10.27am | 20 minutes |
